# Application of a Total Pressure Sensor in Supersonic Flow for Shock Wave Analysis Under Low-Pressure Conditions

**DOI:** 10.3390/s25206291

**Published:** 2025-10-10

**Authors:** Michal Bílek, Jiří Maxa, Pavla Šabacká, Robert Bayer, Tomáš Binar, Petr Bača, Jiří Votava, Martin Tobiáš, Marek Žák

**Affiliations:** 1 Faculty of Electrical Engineering and Communication, Brno University of Technology, Technická 10, 616 00 Brno, Czech Republic; 2 Institute of Scientific Instruments of the CAS, Královopolská 147, 612 64 Brno, Czech Republic; 3 Faculty of AgriSciences, Mendel University in Brno, Zemědělská 1665/1, 613 00 Brno, Czech Republic

**Keywords:** Ansys Fluent, aperture, CFD, differentially pumped chamber, ESEM, low pressure, nozzle, pitot sensors, shock wave

## Abstract

This study examines the design and implementation of a sensor developed to measure total pressure in supersonic flow conditions using nitrogen as the working fluid. Using a combination of absolute and differential pressure sensors, the total pressure distribution downstream of a nozzle—where normal shock waves are generated—was characterized across a range of low-pressure regimes. The experimental results were employed to validate and calibrate computational fluid dynamics (CFD) models, particularly within pressure ranges approaching the limits of continuum mechanics. The validated analyses enabled a more detailed examination of shock-wave behavior under near-continuum conditions, with direct relevance to the operational environment of differentially pumped chambers in Environmental Scanning Electron Microscopy (ESEM). Furthermore, an entropy increase across the normal shock wave at low pressures was quantified, attributed to the extended molecular mean free path and local deviations from thermodynamic equilibrium.

## 1. Introduction

The present manuscript addresses the issue of vacuum-pumped chambers in an Environmental Scanning Electron Microscope (ESEM) under conditions of supersonic flow at low pressures. This supersonic flow arises from the unique configuration of the ESEM, which incorporates, between the tube and the specimen chamber, a differentially pumped chamber. This arrangement enables the maintenance of a large pressure gradient through a cascade of stages, achieving a final pressure ratio of up to 2000 Pa:0.01 Pa between the tube and the specimen chamber. In particular, the ESEM contains, among other elements, a differentially pumped chamber and a specimen chamber, separated by a small aperture equipped with a nozzle, which is the focus of the present study. Due to the large pressure differences between the individual chambers, the gas flow through the nozzle is accelerated to velocities corresponding to Mach numbers greater than unity, resulting in the formation of both oblique and normal shock waves inside the nozzle. The characteristics of these shock waves significantly affect the scattering of the primary electron beam and form an integral part of the broader research in the field of ESEM [1]. The work described herein is conducted at the Institute of Scientific Instruments of the Czech Academy of Sciences (Brno, Czech Republic) by the research team of Vilém Neděla, in collaboration with the Department of Electrical and Electronic Engineering at the Faculty of Electrical Engineering and Communication, Brno University of Technology (Brno, Czech Republic). The ESEM was developed to overcome the stringent vacuum requirements of conventional electron microscopes, thereby enabling the observation not only of conductive and semiconductive samples [2,3,4], but also of non-conductive specimens in their native in situ environments without the need for conductive coating [5,6,7]. As outlined above, the shock waves generated within the nozzle have a pronounced influence on electron beam scattering. The type and shape of these shock waves can be influenced, among other factors, by modifying the geometry and divergence angle of the nozzle. While this phenomenon has been relatively well described for atmospheric pressure conditions, its behavior under the low-pressure conditions characteristic of ESEM operation remains insufficiently explored.

**Figure 1:**
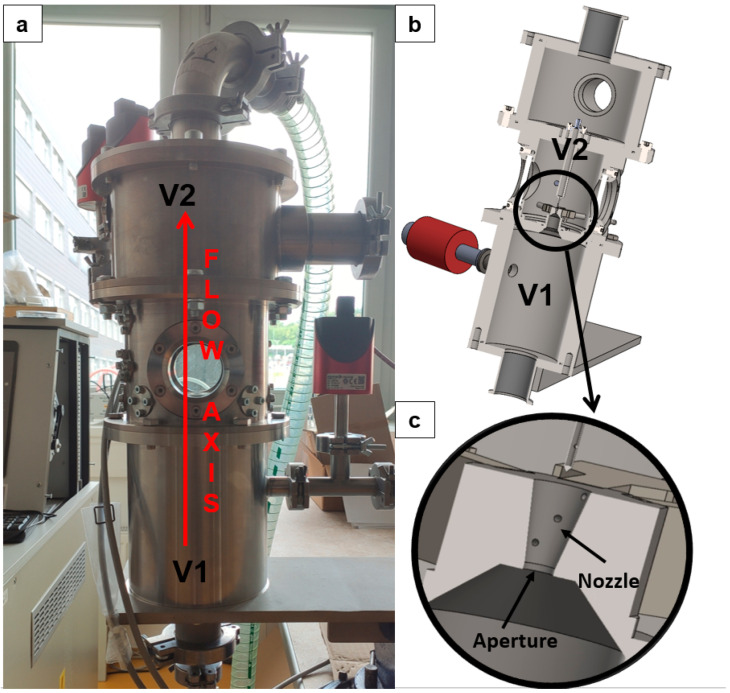
Experimental chamber: real experimental chamber (**a**), 3D solid model (**b**), zoomed area of aperture with nozzle (**c**).

For this reason, an experimental chamber was constructed at the ISI CAS to investigate supersonic flow at low pressures under conditions simulating those in the ESEM. The apparatus consists of two chambers separated by a small conical aperture incorporating a precisely engineered nozzle. In Figure 1c, only a schematic 3D model without edge rounding is shown. In practice, the transition between the aperture and the nozzle is rounded, which was also considered in the CFD analyses. A sharp edge at the transition between the aperture and the nozzle would affect the flow in the initial section of the nozzle. The chamber designated as V1 simulates the specimen chamber in the ESEM, while chamber V2 represents the differentially pumped chamber in the ESEM (Figure 1). In this configuration, the aperture with the nozzle separates two regions with a significant pressure ratio of approximately 10:1. As part of the broader research program aimed at characterizing flow behavior under low-pressure conditions—where the value of the Reynolds number, which determines the ratio of inertial to viscous forces differs substantially from that at atmospheric pressure—the investigation includes the mapping of state variables along the flow axis within the nozzle, downstream of the nozzle, and along the nozzle wall. This mapping is performed using both pressure and temperature sensors. The wall pressure measurements within the nozzle have been reported in [8].

This research serves to refine CFD simulations, as the flow characteristics downstream of the nozzle are highly complex, exhibiting steep gradients. Experimental measurements obtained from sensors are therefore essential to verify the appropriate CFD configuration capable of capturing all relevant physical phenomena [9]. At present, the investigation focuses on mapping the distribution of static and total pressure along the flow axis. Upon completion of the study, the acquired data will also allow for the evaluation of the Mach number distribution.

This paper focuses on the optimization of the sensor apparatus configuration for experimental total pressure measurements using a modified probe, as well as the refinement of CFD simulations for this quantity, and the evaluation of preliminary results within a selected range of pressures, all with a constant pressure ratio of 10:1.

In this contribution, results will be presented for total pressure measurements along the flow axis downstream of the nozzle, obtained using a Pitot tube, for pressure conditions in chamber V1 of 109,000 Pa, 80,000 Pa, 65,000 Pa, and 40,000 Pa. Both the experimental setup and the CFD simulations employed nitrogen, modeled as a real gas using thermophysical properties from NIST rather than assuming ideal-gas behavior. In NIST, the temperature dependence of dynamic viscosity, thermal conductivity is implemented according to Sutherland’s law.

## 2. Methodology

### 2.1. Pitot Tube

**Figure 2:**
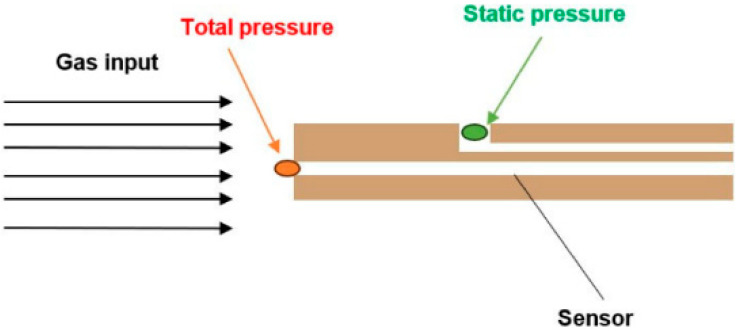
Pitot tube method configuration.

When measuring pressures using Pitot tubes, a pressure sensor is inserted into the flow axis. In general, this method allows for the simultaneous measurement of both total and static pressures—total pressure being captured at the probe’s frontal opening and static pressure at its lateral port, positioned at a sufficient distance from the tip (Figure 2). In our case, however, the experimental chamber simulating ESEM conditions does not permit the simultaneous acquisition of total and static pressures due to the small dimensions of the chamber [10].

**Figure 3:**
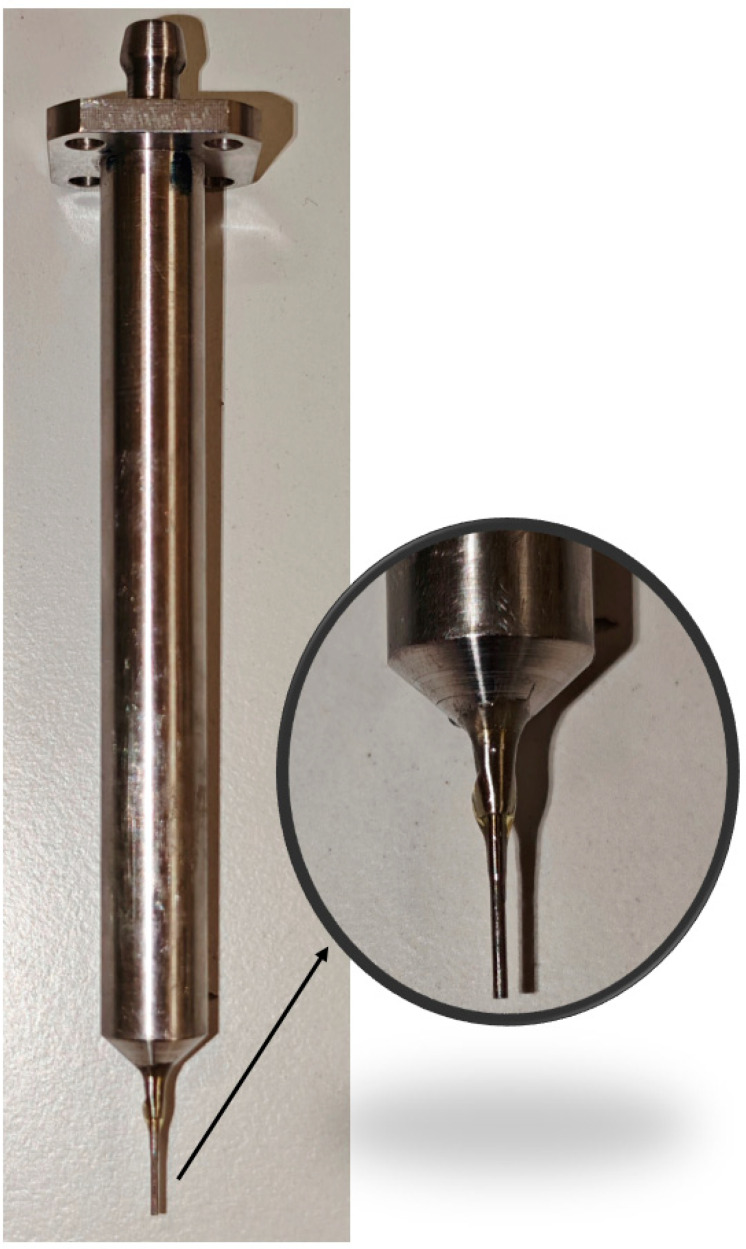
Total Pressure sensor based on Pitot tube.

Consequently, an independent measurement approach was employed, whereby two Pitot tube-based sensors (Figure 3) were utilized within the confined volume of the experimental chamber to examine the low-pressure region. The resulting data were subsequently used to calibrate the CFD model, facilitating comprehensive mapping of the nozzle outflow into free space and enabling the characterization of entropy across shock waves under low-pressure conditions [11].

In parallel, the potential use of Kiel probe was considered. While it also measures the total pressure, it is more appropriate for flows with angular deflection, which does not apply in the present case. A Pitot tube was therefore selected, as it provides a simpler and sufficient solution under well-aligned flow conditions. Strict tolerances were imposed, followed by inspection and fine adjustment to ensure accurate coaxial alignment of the probe with the flow axis. Furthermore, the blockage ratio of the Pitot tube was evaluated, as determined by the commonly employed Equation (1).

(1) $$Blockage\;ratio=\frac{A_{probe}}{A_{flow\;channel}}$$

where *A* is area.

**Figure 4:**
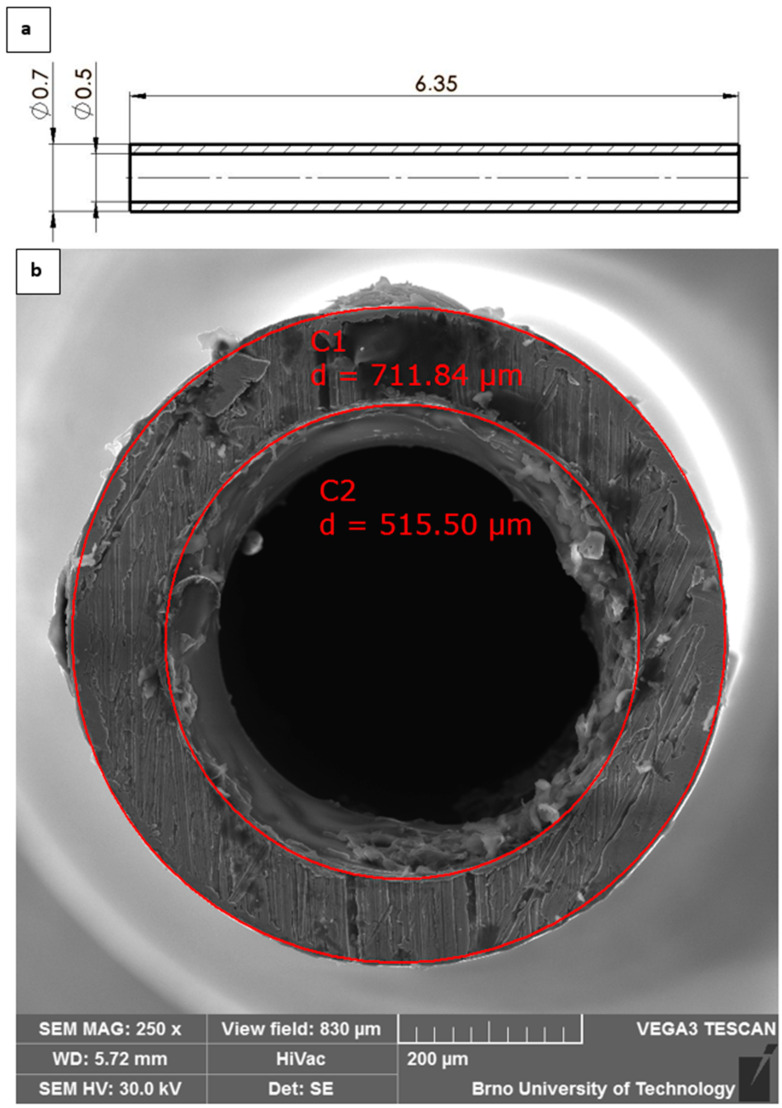
Total Pressure sensor based on Pitot tube—scheme with dimensions [mm] (**a**) and real probe diameters (**b**).

For these initial analyses, a simplified Pitot tube design was employed (Figure 4), featuring a blockage ratio of 0.008%, from which the first adequate results were obtained for characterizing shock-wave structures and flow behavior under low-pressure conditions. In subsequent work, attention will be directed toward reducing the blockage ratio by adopting a conical Pitot tube geometry with a 60° apex angle.

In general, when a sensor with a flat tip is inserted into the flow axis, the flow pattern in front of the sensor face varies depending on the velocity and type of flow [10]. These regimes can be classified into three mathematical categories: incompressible flow, subsonic compressible flow, and supersonic compressible flow.

#### 2.1.1. Incompressible Flow Regime

In the case of incompressible flow, the flow velocity is less than 30% of the speed of sound. Under these conditions, the equation for calculating the total pressure (Equation (2)) is derived from the simple relationship in which the total pressure equals the sum of the dynamic and static pressures (Figure 2).

(2) $$p_{t}=\frac{1}{2}{\rho v}^{2}+p_{stat}$$

where *p_t_* is total pressure, *ϱ* is density, *v* is velocity, and *p_stat_* is static pressure.

#### 2.1.2. Subsonic Compressible Flow Regime

**Figure 5:**
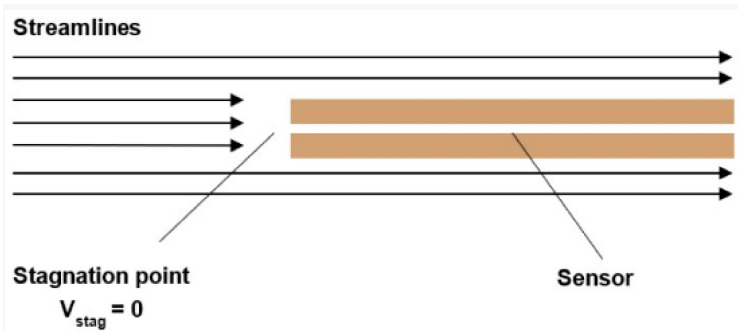
Subsonic compressible flow regime.

For velocities greater than 30% of the speed of sound, the flow falls within the subsonic compressible regime. In this regime, the velocity calculation incorporates the dimensionless Mach number (Equation (3)). The subsonic compressible regime is thus considered for flow velocities from 0.3 to 1 Mach, during which the gas along the Pitot tube is compressed up to the stagnation point. Consequently, the pressure measured at the sensor tip is no longer the total pressure, but rather the stagnation pressure (Figure 5). In this case, the velocity calculation (Equation (4)) additionally involves the specific heat ratio (*γ*) (Equation (5)).

(3) $$M=\frac{v}{c}$$

where *M* is Mach number, *v* is velocity, and *c* is speed of sound in given ambient.

(4) $$v=\sqrt{\frac{2\gamma }{\gamma -1}\frac{p_{static}}{p_{static}}\left[{\left(\frac{p_{stag}}{p_{static}}\right)}^{\left(\frac{\gamma -1}{\gamma }\right)}-1\right]}$$

where *v* is velocity, *γ* is the specific heat ratio, *p_static_* is static pressure, *ϱ_static_* is static density, and *p_stag_* is stagnation pressure.

(5) $$\gamma =\frac{C_{p}}{C_{v}}=\frac{c_{p}}{c_{v}}$$

where *C_p_* is heat capacity at constant pressure, *C_v_* is heat capacity at constant volume, *c_v_* and *c_p_* are the respective specific heat capacities.

#### 2.1.3. Supersonic Compressible Flow Regime

**Figure 6:**
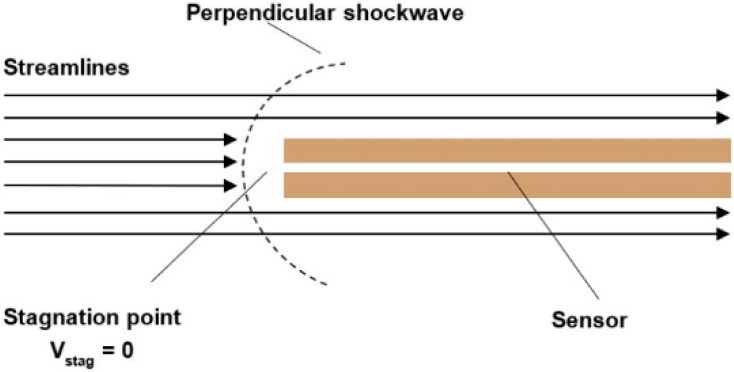
Supersonic compressible flow regime.

For velocities exceeding 1 Mach, the flow regime is classified as supersonic compressible flow. This is the case in our study. In this regime, a normal shock wave forms in front of the pressure sensor tip. The passage of the flow through this shock wave causes a deceleration that is initially non-isentropic to subsonic speeds, followed by an isentropic deceleration to zero velocity at the stagnation point (Figure 6). Consequently, the velocity calculation is considerably more complex than in the previous two cases, as it requires first determining the Mach number iteratively and then solving the ratio of static to stagnation pressure (Equation (6)) [12].

(6) $$\frac{p_{stag}}{p_{static}}=\frac{{\left[\frac{\gamma +1}{2}M^{2}\right]}^{\left(\frac{\gamma }{\gamma -1}\right)}}{{\left[\frac{2\gamma }{\gamma +1}M^{2}-\frac{\gamma -1}{\gamma +1}\right]}^{\left(\frac{1}{\gamma -1}\right)}}=\frac{\gamma +1}{2}M^{2}{\left[\frac{{\left(\gamma +1\right)}^{2}M^{2}}{4\gamma M^{2}-2(\gamma -1)}\right]}^{\left(\frac{1}{\gamma -1}\right)}$$

In this case, it is also necessary to consider the theory of isentropic one-dimensional flow across a normal shock wave (these can be found in [13,14,15]), which relates the flow properties upstream and downstream of the shock. The purpose of this theoretical analysis is to validate the results obtained from the CFD simulations.

The relationship between the Mach number and the total pressure in supersonic nozzle flow is described by the equation for isentropic flow, which holds under the assumption that the flow is adiabatic and frictionless. The total pressure, sometimes also referred to as the stagnation pressure, is the pressure the gas would have if it were isentropically decelerated to zero velocity (stagnation). In the ideal case of isentropic flow in a nozzle, where no energy losses occur (e.g., due to friction or shock waves), the total pressure remains constant. The relationship between the static pressure, total pressure, and Mach number is given by Equation (7).

(7) $$\frac{P_{t}}{P_{stat}}={\left(1+\frac{\gamma -1}{2}M^{2}\right)}^{\frac{\gamma }{\gamma -1}}$$

where *γ* is the specific heat ratio (adiabatic index), which is constant for an ideal gas. For nitrogen, the working gas in our case, a value of *γ* = 1.4.

From Equation (7), it follows that in supersonic flow (*M* > 1), the ratio of total pressure to static pressure increases with the Mach number. Since the total pressure remains constant in isentropic flow, this implies that the static pressure must decrease as the Mach number increases. This phenomenon is fundamental to the design of supersonic nozzles: for the gas to reach supersonic velocity, it must expand into a region of increasing cross-sectional area, accompanied by a rapid drop in static pressure. This behavior is in stark contrast to that of subsonic flow, where velocity decreases as the cross-sectional area increases.

It is important to emphasize that Equation (7) applies only to isentropic flow. In practice, supersonic flows typically involve the presence of shock waves. A shock wave is defined as a discontinuity in the flow where the flow properties undergo an almost instantaneous jump change. Unlike conventional isentropic processes, which involve gradual and lossless compression, a shock wave is an intrinsically irreversible phenomenon. This fact has a fundamental impact on the energy balance of the flow. While, for example, in a compression wave the smooth change in flow properties would occur without losses in an isentropic manner, such an ideal process is not physically realizable in practice. A shock wave arises precisely because a continuous transition at supersonic velocities is physically impossible, and its behavior is governed by the Rankine–Hugoniot equations.

The Rankine–Hugoniot equations describe the relationships between the properties of a fluid on either side of a shock wave—a sudden, discontinuous transition characterized by abrupt changes in pressure, density, temperature, and flow velocity. These equations are derived from the conservation laws applied across this discontinuity.

The continuity equation states that the mass flow rate into the shock wave must equal the mass flow rate out of it. The momentum equation describes the balance of forces—specifically pressure and momentum—on both sides of the shock wave. The energy equation states that the total energy—comprising internal, kinetic, and pressure energy—is conserved on both sides of the shock wave.

These three equations (continuity, momentum, and energy equations) constitute a system that describes the discontinuous changes in pressure, density, and temperature occurring across a shock wave.

### 2.2. Ansys Fluent Settings

The CFD simulations were conducted using the Ansys Fluent system (Canonsburg, PA, USA), which solves the Navier–Stokes equations and is exclusively employed for describing flow behavior within continuum mechanics [16,17]. Ansys Fluent offers a range of configuration options, from settings suited for simple problems to those required for highly complex simulations. It is essential to configure Ansys Fluent appropriately to match the complexity of the task, ensuring neither underestimation nor overestimation of the problem’s demands [18,19].

For the CFD model configuration presented in this study, the Density-Based solver was employed, considering the results of experimental measurements. This solver simultaneously solves the conservation equations for mass, momentum, and energy [20,21]. It was selected because this approach is ideal for supersonic flows with shock waves, as it effectively captures the strong nonlinearities and rapid density variations associated with such phenomena. Additionally, the Coupled solver was chosen due to its faster and more stable convergence, achieved through the coupled solution of the governing equations. This feature makes it particularly suitable for supersonic flows involving shock waves. Based on this setup, flux interpolation of the Rhie-Chow distance-based type was implemented for calculating fluxes at cell faces. This method, which relies on distance weighting, helps prevent pressure oscillations and provides a more stable and accurate solution on unstructured computational meshes (e.g., triangles, etc.).

For gradient calculation, the Second-Order scheme was selected as it best captures shock waves typical of compressible flow. The fundamental principle of this approach is that the variable value at the cell face (e.g., density) is computed via linear extrapolation from the cell centroid from which the flow originates [22]. This method provides higher accuracy compared to the First-Order scheme, which considers only the value from a single cell. Furthermore, to ensure accurate gradient representation, the Warped-Face Gradient Correction option was employed. This technique enhances the precision of gradient calculations on imperfect mesh cells by applying a corrective algorithm. The standard finite volume method assumes cell faces are planar, which can lead to errors in gradient computations for complex geometries such as the one in this study [23].

For turbulence modeling, the SST (Shear Stress Transport) k–omega model was selected, which combines the k–omega model for resolving the boundary layer region—including flow separation—with the k–epsilon model for the free stream. This blending approach is highly appropriate for the present case and represents an optimal tool that merges the high accuracy of the k–omega model with the robustness of the k–epsilon model. Additionally, the viscous heating option was enabled in the turbulence model. This adds a term to the energy equation accounting for the conversion of mechanical energy into thermal energy due to viscosity. Viscous friction between fluid layers generates heat, which can locally increase the temperature, thereby affecting fluid properties such as density and dynamic viscosity. This feature was incorporated because of the compressible, high-velocity flow conditions, where viscous heating significantly influences the overall temperature field. The Compressibility Effects option was also activated to adapt the turbulence model for high-speed compressible flows. This feature is crucial because turbulence characteristics change at high Mach numbers, where density, temperature, and pressure fluctuate strongly, impacting the calculation of turbulent viscosity. The option accounts for turbulence energy dissipation in highly compressible regions, such as shock waves. Without this correction, the model might overpredict turbulence production, leading to inaccurate results. Moreover, the impact of the Production Kato-Launder function was tested. This function aims to limit excessive production of turbulent kinetic energy (k), which the SST k–omega model tends to overpredict in regions of high strain, potentially distorting results. It achieves this by replacing the strain rate tensor in the turbulence production term with the vorticity tensor. In the present study, this correction does not significantly affect the results but will be applied in future analyses of flow around Pitot tubes. A similar function, the Production Limiter, also restricts excessive turbulent kinetic energy production that may occur in flow regions such as stagnation points or zones with high shear stress. This prevents overestimation of turbulent viscosity and heat transfer. Like the Kato-Launder correction, the Production Limiter will be relevant primarily in the analysis of flow around Pitot tubes [24,25].

As the final configuration, the wall boundary condition was set to adiabatic due to the high flow velocities and the negligible heat transfer between the fluid flow and the wall [26]. Total temperature used for all variants was 297.15 K.

Inflation layers were applied in the regions of the aperture, nozzle, Pitot tube, and their vicinity, starting directly from the wall. A total of ten layers were specified. The thickness of the first layer was determined using the relation for evaluating the size of the first cell adjacent to the wall, based on the parameters *y^+^*, dynamic viscosity (*µ*), wall shear stress velocity (*U_τ_*), and density (*ϱ*). The first cell height was chosen so that *y^+^* did not exceed a value of 1. For the initial computation, this thickness was estimated using assumed values, and was subsequently adjusted according to the results obtained from the first simulations.

### 2.3. Experimental Measurement Settings

**Figure 7:**
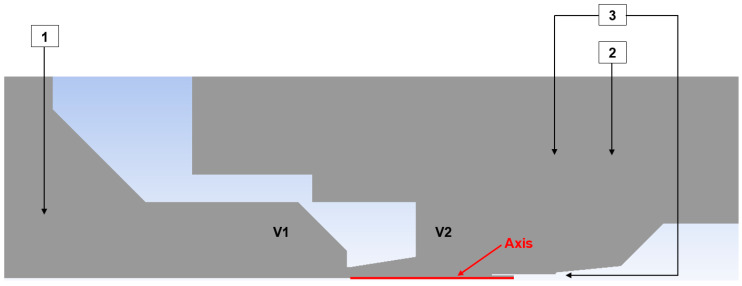
Sensors configuration for total pressure experimental measuring.

The total pressure measurement was conducted, as described above, using the Pitot tube method for total pressure measuring. Figure 7 presents a 2D axisymmetric schematic layout of the pressure sensors used in the experimental setup.

Pressure sensors 1 and 2 are absolute pressure sensors [27], while sensor 3 is a differential pressure sensor [28]. The latter was chosen to improve measurement accuracy due to its lower error associated with a smaller measurement range.

Specifications of the individual sensors are provided in Table 1.

Based on the configuration shown in Figure 6, for each pressure variant (109,000 Pa; 80,000 Pa; 65,000 Pa; and 40,000 Pa), the pressure ratio between chambers V1 and V2 was established using the absolute pressure sensors listed in Table 1. This pressure ratio was approximately 10:1; for example, in the 109,000 Pa variant, the pressure in chamber V1 was 109,000 Pa, while in chamber V2 it was 10,900 Pa. Similar ratios applied for the other variants. A differential pressure sensor was inserted into the Pitot tube used for total pressure measurement [29,30]. The results obtained from this measurement will subsequently be compared with CFD analyses.

## 3. Results

**Figure 8:**
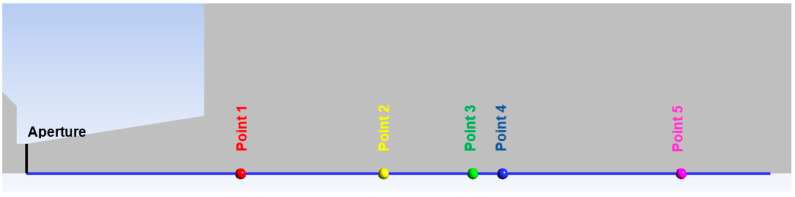
Point positions for experimental measuring with the path (blue line) for variables distribution.

Table 2 presents the distances of the measured points from the aperture for total pressure measuring using a Pitot tube sensor. The points indicated in Figure 8 show the locations where the tip of the Pitot tube is positioned for the total pressure measurement in this specific case. Figure 8 also shows the path (blue line) along which the Mach number, total pressure, and entropy are plotted in subsequent analyses.

Results of the experimental measuring performed using the constructed sensor and attached probes (Figure 7) are presented in Table 3. The data includes the absolute pressure in chamber V1 measured by Probe 1 (Table 1), the absolute pressure in chamber V2 measured by Probe 2, and the differential pressure between chamber V2 and the total pressure probe measured by Probe 3. The total pressure value is obtained from the difference between Probe 3 and Probe 2, which is then measured by the total pressure sensor at the selected points (Figure 8).

**Figure 9:**
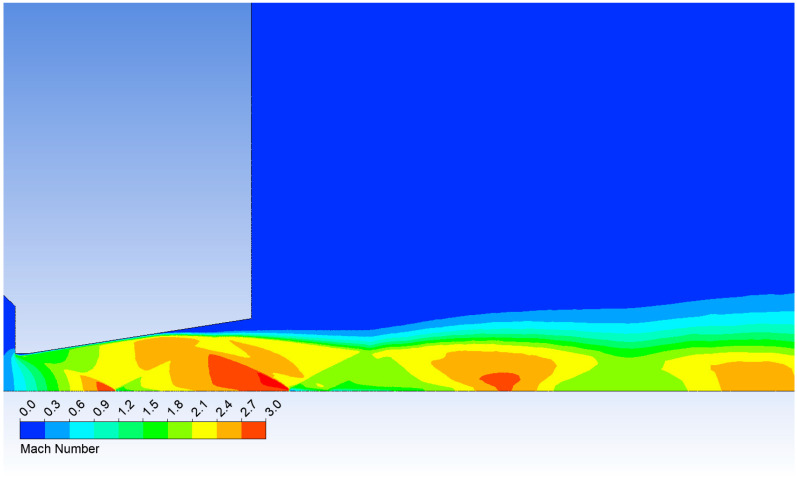
Mach number layout in the nozzle for variant of 109,000 Pa.

Figure 9 presents the Mach number distribution in the nozzle and the downstream region for the 109,000 Pa variant to illustrate the flow characteristics. Shock-wave locations and flow separation on the nozzle wall are evident, resulting from the pressure ratio across the nozzle that produces underexpanded flow within the nozzle.

Eight repeated measurements of pressure differentials were carried out at a distance of 12 mm for the variant of 109,000 Pa, and the averaged values served as the basis for subsequent analysis. To evaluate the accuracy of the measurements, the Standard Error of the Mean (SEM) was calculated. The SEM, defined as the standard deviation of the sampling distribution of the mean, indicates how far the sample mean is expected to deviate from the true population mean.

The calculation followed Equation (8), and the corresponding results are summarized in Table 4.

(8) $$SEM=\frac{\sigma }{\sqrt{n}}$$

Based on these eight repeated measurements (Table 4), a rigorous uncertainty budget following ISO/GUM was also established. This was calculated using Equations (9)–(16), and the results are presented in Table 5.

Measurement model:

(9) $$y=f\left(x_{1},x_{2},\ldots ,x_{N}\right)$$

Sensitivity coefficient:

(10) $$c_{i}={\left.\frac{\partial f}{\partial x_{i}}\right|}_{x_{1},\ldots ,x_{N}}$$

Covariance between inputs:

(11) $$cov\left(x_{i},x_{j}\right)=r_{ij}\;u\left(x_{i}\right)\;u{(x}_{j})$$

where *r_ij_* is correlation coefficient ($-1\;\leq \;r_{ij}\;\leq \;1)$.

Combined standard uncertainty:

(12) $$u_{c}^{2}\left(y\right)=\sum_{i=1}^{N}c_{i}^{2}u^{2}\left(x_{i}\right)+2\sum_{i<j}c_{i}c_{j}\mathrm{cov}\left(x_{i},x_{j}\right)$$

Effective degrees of freedom (Welch–Satterthwaite):

(13) $$\nu_{eff}=\frac{u_{c}^{4}\left(y\right)}{\sum_{i=1}^{N}\frac{c_{i}^{4}u^{4}\left(x_{\dot{i}}\right)}{\nu_{i}}}$$

Expanded uncertainty:

(14) $$U=k\cdot u_{C}\left(y\right)$$

where *k* is coverage factor z t-distribution:

(15) $$k=t_{1-\alpha ∕2,\nu_{eff}}$$

Measurement result (with 95% confidence interval):

(16) $$y=\hat{y}\pm U$$

**Figure 10:**
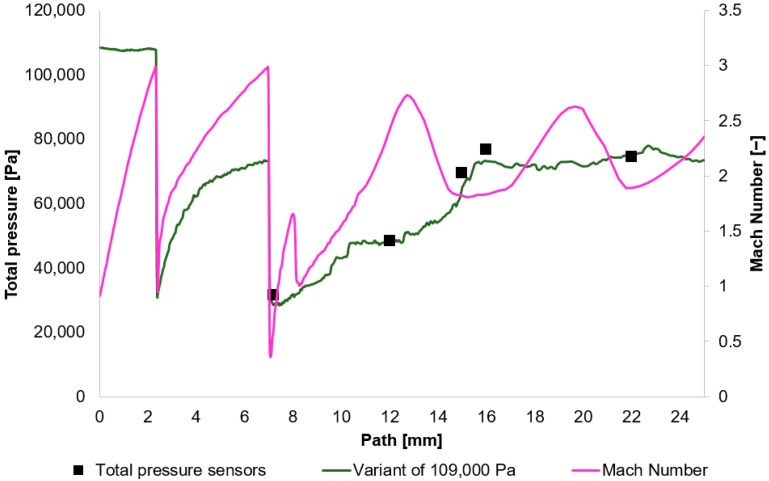
Total Pressure and Mach number distribution on path for variant of 109,000 Pa.

**Figure 11:**
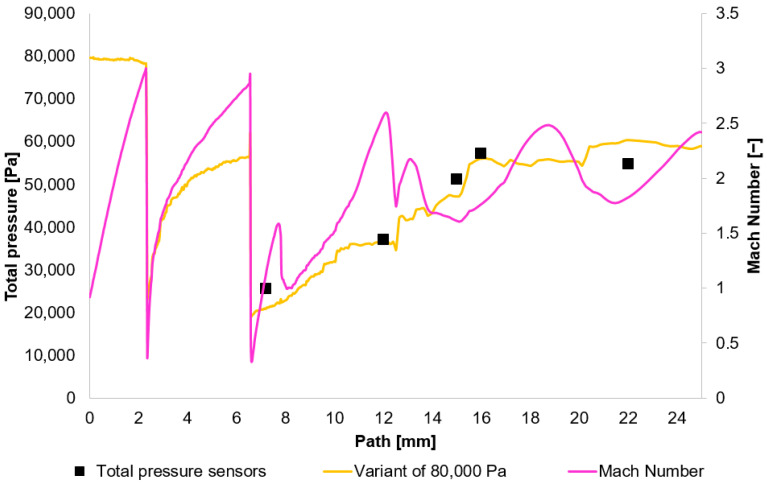
Total Pressure and Mach number distribution on path for variant of 80,000 Pa.

**Figure 12:**
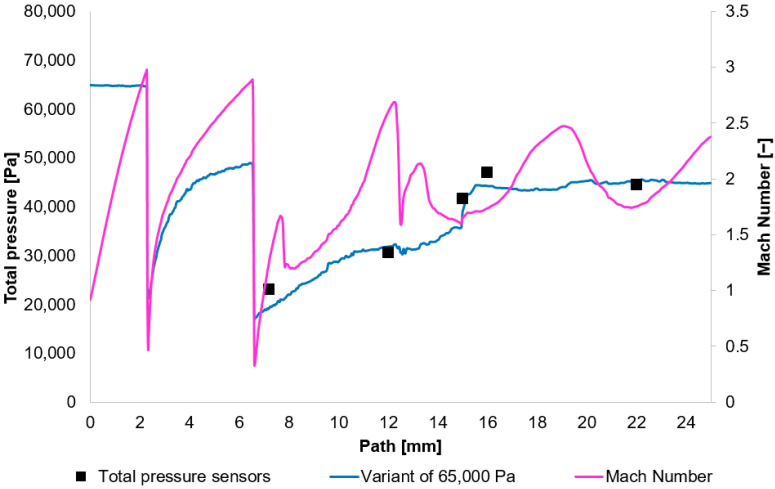
Total Pressure and Mach number distribution on path for variant of 65,000 Pa.

**Figure 13:**
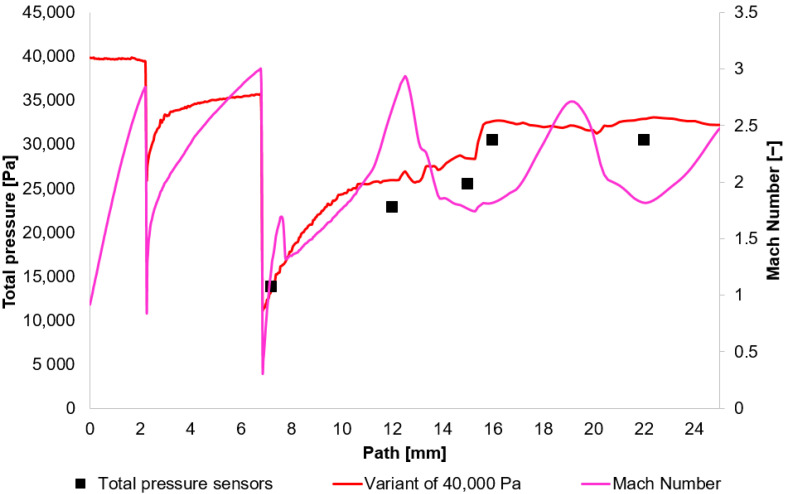
Total Pressure and Mach number distribution on path for variant of 40,000 Pa.

The results obtained from the experimental measuring were compared with the results acquired using the Ansys Fluent system. Based on these results, Figure 10, Figure 11, Figure 12 and Figure 13 initially plot the Mach Number and total pressure for each variant, along with the total pressure values measured experimentally using a Pitot tube. Table 6 then evaluates the error according to Equation (17).

(17) $$\frac{Experiment-CFD}{Experiment}\times 100$$

In Figure 10, Figure 11, Figure 12 and Figure 13, sharp drops in the Mach number below a value of 1 Mach indicate the location of normal shock waves. During this process, there is a noticeable loss of total pressure, which is also evident in the presented graphs (Section 2.1.3).

The amount of total pressure loss is dependent on the strength of the shock wave, which is directly proportional to the Mach number in front of the wave. The higher the Mach number in front of the shock wave, the greater the total pressure loss. However, since the Mach number is directly determined by the total pressure ratio between the nozzle’s inlet and outlet, it is logical that in our case, with the same pressure drop of approximately 10:1, the Mach number at the shock wave is essentially the same.

**Figure 14:**
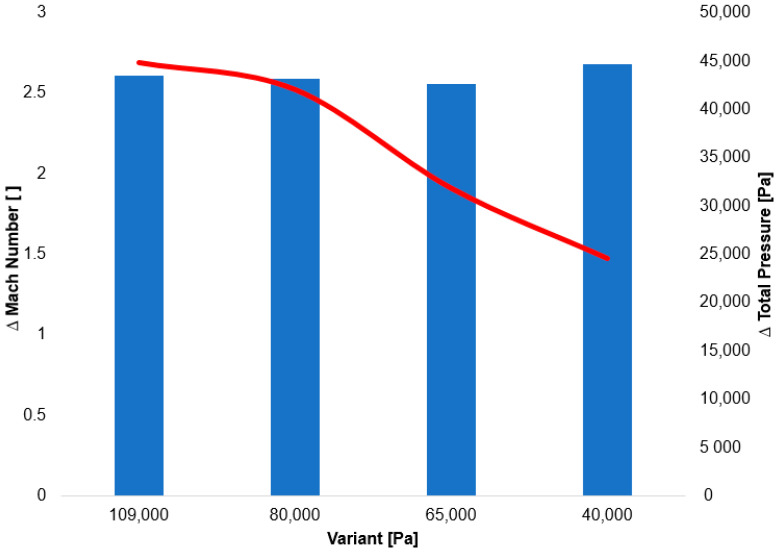
Summary graph of the decrease in total pressure on the second shock wave.

The distinct total pressure drop curves for each variant, confirmed by experimental measurements, indicate a change in the value of Reynolds number at lower pressures (Figure 14).

The greater drop in total pressure at lower pressure is therefore due to a significantly lower value of Reynold number. Down to a pressure of approximately 133 Pa, viscosity is not dependent on pressure, while the values of inertial forces decrease with dropping pressure. For gases, dynamic viscosity is primarily dependent on temperature, not pressure, and remains relatively constant. However, the gas density is directly proportional to pressure. It follows that as pressure decreases, density also decreases, and therefore, the Reynolds number drops. A decrease in the Reynolds number signifies that the relative importance of viscous forces increases compared to inertial forces.

The following research was conducted on the second shock wave, located approximately 7 mm from the aperture (Figure 8). From the graphs in Figure 10, Figure 11, Figure 12 and Figure 13, the results for Mach Number and total pressure both in front of and behind the shock wave were plotted in a summary graph (Figure 14) and a table (Table 7). The results confirm the earlier statement that as pressure decreases, the magnitude of the total pressure value at the second shock wave drops more significantly. The reason for this is the influence of low pressure with a longer mean free path of the molecule, which is discussed further below.

Given the facts mentioned above, the core of the problem is the relationship between the drop in total pressure and thermodynamic irreversibility. Every irreversible process is associated with energy dissipation, i.e., the conversion of organized mechanical flow energy into disorganized internal energy (heat), which is manifested by an increase in entropy, *Δs*. The drop in total pressure is a direct measure of this thermodynamic irreversibility. The greater the irreversibility, the greater the increase in entropy and the greater the drop in total pressure.

A key relationship exists between the increase in specific entropy and the total pressure ratio, which can be expressed by Equation (18):

(18) $$\Delta S=S_{2}-S_{1}=-R.l_{n}\left(\frac{p_{02}}{p_{01}}\right)$$

where *Δs* is the specific entropy, *r* is the specific gas constant, and *p_0_*_1_ a *p_0_*_2_ are the total pressures before and after the shock wave.

The specific gas constant *r* is the ratio of the universal gas constant *R* to the molar mass *M* of a given gas. The universal gas constant *R* has a value of 8.31446 J·mol^−1^·K^−1^. The molar mass of nitrogen (N_2_) is approximately 28.0134 g.mol^−1^. Therefore, the specific gas constant for nitrogen is 296.8 J·kg^−1^·K^−1^.

**Figure 15:**
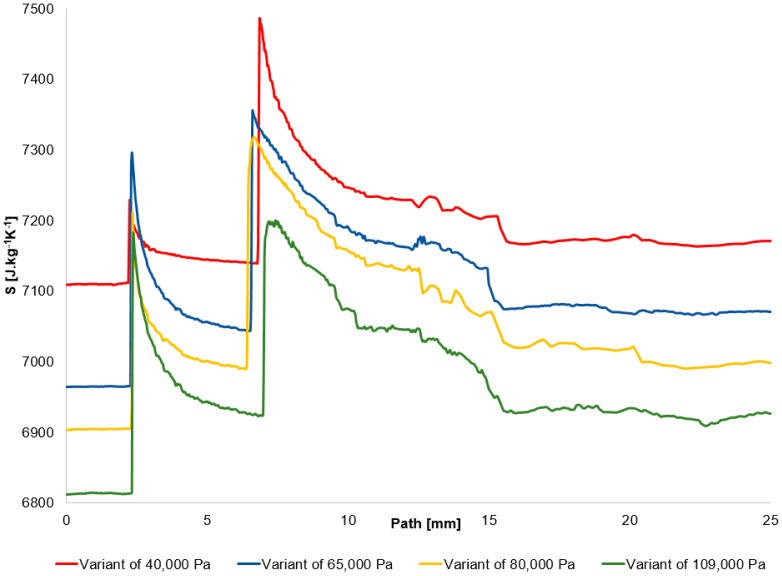
Entropy distribution on the path.

The graph in Figure 15 shows the entropy profile along the studied path from the Ansys Fluent system.

In the following table (Table 8), the verification of the entropy change at the second shock wave is performed using both parts of Equation (18). This includes the verification of the entropy change based on the total pressure change at the shock wave, as depicted in the graph in Figure 15, as well as the entropy change itself from the graph in Figure 14.

The results are essentially identical and show that as pressure decreases, the change in entropy, and thus the loss at the shock wave, increases.

It should be noted that a shock wave is not an ideal, infinitely thin discontinuity, but possesses a finite thickness. This thickness is directly related to the mean free path of the molecules *λ*, which is the average distance a molecule travels between collisions. The mean free path is inversely proportional to pressure. Therefore, as pressure decreases, the mean free path increases, and the physical thickness of the shock wave also increases. This makes the shock wave a more gradual transition rather than a sharp jump.

The classic thermodynamic equation mentioned above (Equation (18)) states that the increase in entropy across a shock wave is directly linked to the decrease in total pressure. The greater the loss of total pressure—that is, the lower the ratio of *p*_02_ to *p*_01_—as is the case in our study, the greater the increase in entropy and the irreversibility of the process. When the flow enters a region of rarefied gas, energy dissipation actually increases. This increased dissipation leads to a greater drop in total pressure, which is manifested as a lower *p*_02_ to *p*_01_ ratio. This phenomenon is caused by the increase in the mean free path of the molecules as the pressure decreases, making the shock wave structure thicker. This thicker transition is a physical consequence of the violation of local thermodynamic equilibrium, which paradoxically leads to greater, not smaller, losses.

Finally, an outline for future research can be provided. The static pressure profile will be processed in a similar manner using Pitot tubes with side static pressure taps for the same pressure ratios and conditions.

This comprehensive research, of which this paper is a small part, serves, among other things, to investigate the field of Environmental Scanning Electron Microscopy (ESEM) and the effect of primary electron beam scattering as it passes through a differentially pumped chamber. This chamber ensures that the primary electron beam enters the high-pressure specimen chamber from a low-pressure region. As previously stated, a normal shock wave at low pressure has different properties: the mean free path of molecules increases, which leads to the physical “thickening” of the shock wave, resulting in a greater increase in entropy. This indicates the “most efficient” energy losses, which may influence scattering in the given area.

**Figure 16:**
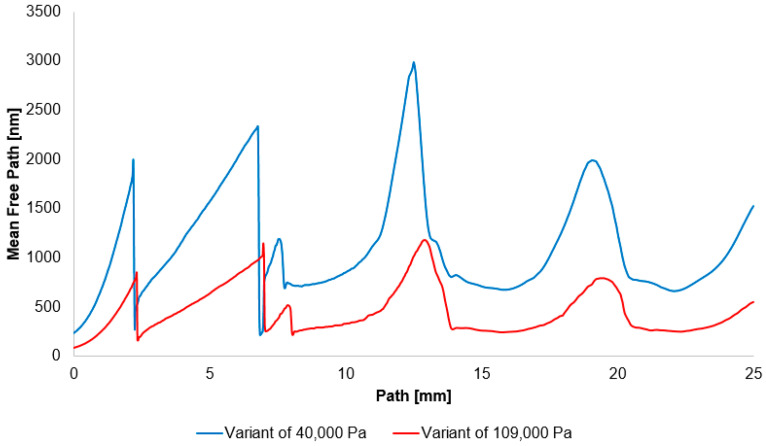
Mean free path distribution on the path for variant of 40,000 Pa and 109,000 Pa.

It has been demonstrated that in the shock-wave region, the mean free path increases significantly at reduced pressure compared to standard atmospheric conditions (Figure 16).

(19) $$\lambda =\frac{1}{\sqrt{2}\pi d^{2}n}=\frac{1}{\sqrt{2}\pi d^{2}\frac{P}{R\;T\;M}}=\frac{R\;T}{\sqrt{2}\pi d^{2}P}$$

where *d* is diameter of the molecule, *n* is number density, *M* is Molecular weight, *P* is static pressure, and *T* is static temperature.

The mentioned effects have a direct impact on the electron beam. A shock wave is a zone with a sharp increase in gas density. As the electron beam passes through it, it undergoes concentrated and increased scattering in this area. This disrupts the beam’s collimation and reduces its intensity. Similarly, in the high-density gas zone, the energy losses of electrons due to collisions with molecules (both elastic and inelastic) sharply increase. Because the shock wave is thicker at low pressure, these interactions do not occur in a discrete jump but in a smoother, more measurable transition. This fact can be experimentally utilized to measure the density profile of the shock wave using an electron beam, which will also be a further part of the research.

The increase in entropy within a shock wave is a thermodynamic measure of an irreversible process where a portion of the organized flow energy is converted into disorganized heat. Electron scattering and energy losses are molecular interactions (collisions) that cause this macroscopic entropy. The more collisions that occur, the more energy is dissipated, leading to a greater increase in entropy.

Entropy and electron scattering in a shock wave are mutually dependent. A shock wave with a higher entropy necessarily implies more intense molecular interactions, which in turn leads to greater electron scattering. Therefore, it is valid to state that entropy is the macroscopic, thermodynamic measure of a shock wave’s irreversibility and energy dissipation. Scattering, on the other hand, is the microscopic, quantum manifestation of molecular collisions and energy dissipation, which becomes concentrated in a thin zone due to the rapid increase in density within the wave. Thus, it can be concluded that a shock wave that generates greater entropy inevitably represents a region with more intense and frequent molecular interactions, and consequently, a region with higher electron scattering.

### Experimental Measurement Verification

**Figure 17:**
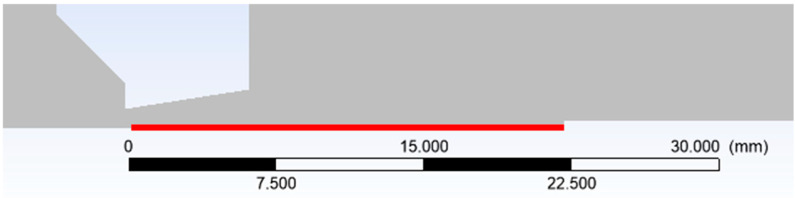
Examined path (red line).

In reference [31], an analysis of the effect of reduced pressure on wall slip in nozzles was conducted. In our case, within the experimental chamber, the slip flow regime is reached at even lower pressures than those considered in the cited study. For the scenario corresponding to the lowest investigated pressure in the chamber above the nozzle (variant 40,000 Pa), a comparison of CFD results was performed with the Pitot tube inserted at a distance of 22 mm from the aperture (Figure 17).

**Figure 18:**
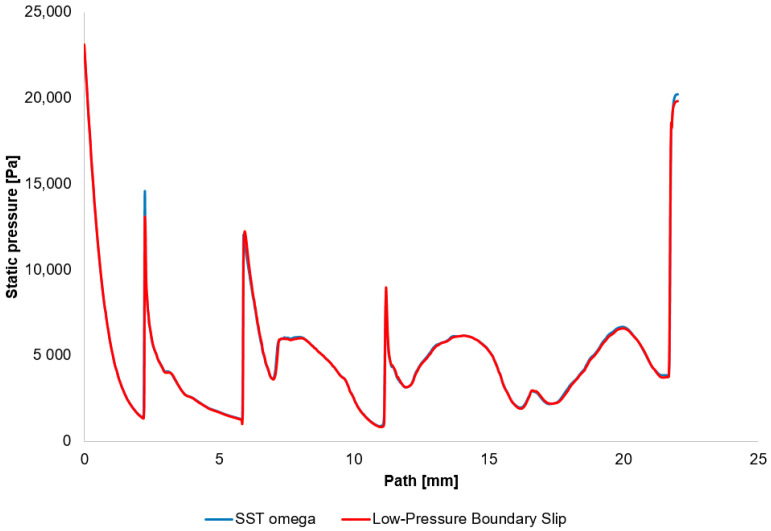
Static pressure distribution on the examined path.

The first variant employed the SST—ω turbulence model with Viscous Heating and Low-Re corrections. In the second variant, the model was configured using Viscous Heating and Low-Pressure Boundary Slip. Both CFD analyses demonstrated essentially identical results in the location of the shock waves (Figure 18).

**Figure 19:**
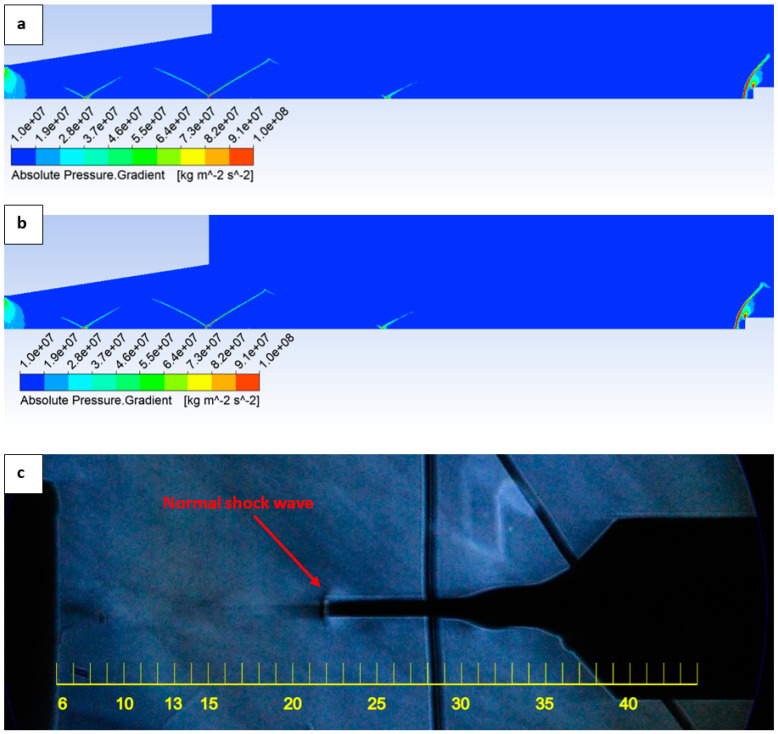
Static pressure layout in the experimental chamber for SST omega variant (**a**), Low-Pressure Boundary Slip variant (**b**) and experimental Schlieren optic method (**c**).

Furthermore, the position of the normal shock wave forming in front of the Pitot tube tip was verified both by CFD analyses and experimentally using the Schlieren method. Both CFD analysis variants (Figure 19a,b) yielded consistent results, indicating that the normal shock wave is located 0.23 mm from the tube tip. The same result was confirmed experimentally using the optical Schlieren technique (Figure 19c).

For verification, an analytical relation (Equation (20)) [32] was also employed, in which the distance of the shock wave is proportional to the radius of the probe tip, and decreases with increasing Mach number.

(20) $$б=\frac{R_{b}}{\sqrt{M_{ꝏ}^{2}-1}}$$

where *б* is the distance of the shock wave from the probe tip, *R_b_* is he radius of the probe tip, and *M_ꝏ_* is the Mach number at the measured location.

Equation (20) is a simplified relation and is generally valid for high Mach numbers. At low supersonic Mach numbers, the ratio *б/R_b_* is larger, and as the Mach number approaches 1, it tends to infinity, placing the shock wave directly at the tube tip. For our case, the same relation yields a value of *б* = 0.23 mm. The dimensions of the probe are provided in Figure 4.

## 4. Conclusions

A sensor based on the Pitot tube principle was successfully designed and constructed to measure total pressure. Using absolute and differential pressure sensors, the total pressure profile at selected points downstream of the nozzle was mapped. Measurements were performed with a pressure ratio of 10:1, which generated supersonic flow and normal shock waves, and were conducted for various pressure conditions within the low-pressure regime. These experimental results were crucial for the verification and refinement of CFD analyses, specifically for simulating the challenging phenomena of supersonic flow in low-pressure environments near the continuum-flow boundary. The validated CFD models now enable a more detailed mapping of shock wave behavior under different pressure conditions approaching this boundary, which is directly relevant to the operating conditions of a differentially pumped chamber in an ESEM. Our findings demonstrate that entropy increases within normal shock waves at low pressures due to the larger mean free path of molecules, leading to a greater local disturbance of thermodynamic equilibrium. This elevated entropy value indicates more intense molecular interactions within the shock wave. The results of this study will serve as a foundational basis for future analyses of the scattering of a primary electron beam by a high-entropy normal shock wave.

## Figures and Tables

**Table 1:** Specification of each pressure sensor.

-	Sensor Name	Scale	Error
1	Pfeiffer CMR 361	110 kPa	±0.2% of the measured value
2	Pfeiffer CMR 362	11 kPa	±0.2% of the measured value
3	DPS 300	25 kPa	±1% Full Scale Output BFSL (above 0.6 kPa)

**Table 2:** Location of the points positions for experimental measuring.

Position	L [mm]
Aperture	0
Point 1	7.2
Point 2	12
Point 3	15
Point 4	16
Point 5	22

**Table 3:** Results of experimental measuring from individual sensors.

Probe 1 V1 [Pa]	Probe 2 V2 [Pa]	Probe 3 Difference [Pa]	Total Pressure [Pa]
Position 7.2 [mm]			
109,000	9051	30,000	39,051
80,000	6692	18,900	25,592
65,000	5481	17,500	22,981
40,000	3385	10,450	13,835
Position 12 [mm]			
109,000	9332	39,100	48,432
80,000	6850	30,200	37,050
65,000	5583	25,000	30,583
40,000	3454	19,400	22,854
Position 15 [mm]			
109,000	9388	60,100	69,488
80,000	6889	44,300	51,189
65,000	5608	36,100	41,708
40,000	3466	22,000	25,466
Position 16 [mm]			
109,000	9392	67,500	76,892
80,000	6894	50,300	57,194
65,000	5610	41,400	47,010
40,000	3462	27,000	30,462
Position 22 [mm]			
109,000	9398	65,000	74,398
80,000	6920	47,800	54,720
65,000	5608	38,898	44,506
40,000	3468	26,988	30,456

**Table 4:** Evaluation of the Standard Error of the Mean (SEM) from Measured Pressure Values.

Measurement Number	Pressure [Pa]
1	48,432
2	48,450
3	48,430
4	48,431
5	48,442
6	48,439
7	48,430
8	48,449
Arithmetic mean [Pa]	48,438
*σ*	8.41
*SEM* [Pa]	2.97

**Table 5:** Rigorous uncertainty budget following ISO/GUM.

Mean (value of the quantity) $\bar{X}$	48,437.88
Sample standard deviation *s*	8.408
Standard uncertainty of the mean (Type A) $u_{c}=u\left(\bar{X}\right)=\frac{s}{\sqrt{n}}$	2.973
Degrees of freedom (Welch–Satterthwaite) $\nu_{eff}=n-1$	7
Expansion factor for 95% $k=t_{0.975,7}$	2.365
Expanded uncertainty (95% confidence) $U=k\cdot u_{C}$	7.03
Final result (95% confidence interval) *y*	48,437.88±7.03

**Table 6:** Comparison of pressure values obtained from experimental measurements with values obtained from the Ansys Fluent system for each variant.

	Experimental Sensor Position from Aperture [mm]				
	7.2	12	15	16	22
109,000 Pa					
Experiment [Pa]	39,051	48,432	69,488	76,892	74,398
CFD [Pa]	31,540	47,962	64,992	73,259	75,264
Rel. Error [%]	19.23382	0.970433	6.470182	4.724809	−1.16401
80,000 Pa					
Experiment [Pa]	25,592	37,050	51,189	57,194	54,720
CFD [Pa]	20,853	36,590	47,170	55,809	60,339
Rel. Error [%]	18.51751	1.241565	7.851296	2.421583	−10.2686
65,000 Pa					
Experiment [Pa]	22,981	30,583	41,708	47,010	44,506
CFD [Pa]	19,286	31,811	38,999	44,236	45,332
Rel. Error [%]	16.0785	−4.0153	6.495157	5.900872	−1.85593
40,000 Pa					
Experiment [Pa]	13,835	22,854	25,466	30,462	30,456
CFD [Pa]	13,499	25,931	28,402	32,658	32,898
Rel. Error [%]	2.428623	−13.4637	−11.5291	−7.20898	−8.01812

**Table 7:** Summary values of the Total Pressure drop on the second shock wave.

Variant	Mach Number in Front of the 2nd Shock Wave	Mach Number Behind the 2nd Shock Wave	Difference	T_2o1_ [Pa]	T_2o2_ [Pa]	Difference	Ratio
109,000	2.98	0.36	2.62	73,262	28,472	44,790	0.389
80,000	2.94	0.33	2.61	61,507	19,050	42,457	0.342
65,000	2.88	0.32	2.56	48,852	17,004	31,848	0.348
40,000	3	0.32	2.68	35,731	11,113	34,618	0.311

**Table 8:** Verification of the entropy change on the second shock wave.

	$\Delta S=-R.l_{n}\left(\frac{p_{02}}{p_{01}}\right)$		$\Delta S=S_{2}-S_{1}$			
Variant	$\frac{p_{02}}{p_{01}}$	∆S	∆S	S_2_	S_1_	Difference
109,000	0.389	280.54	280	7200	6920	0.54
80,000	0.342	318.55	320	7315	6995	−1.45
65,000	0.35	313.20	312	7356	7044	1.03
40,000	0.31	346.65	341	7480	7139	5.65

## Data Availability

The data presented in this study are available on request from the corresponding author.
